# The Management of Clinical Varicocele: Robotic Surgery Approach

**DOI:** 10.3389/frph.2022.791330

**Published:** 2022-03-22

**Authors:** Luigi Napolitano, Savio Domenico Pandolfo, Achille Aveta, Lorenzo Cirigliano, Raffaele Martino, Gennaro Mattiello, Giuseppe Celentano, Biagio Barone, Claudia Rosati, Roberto La Rocca, Gianluca Spena, Lorenzo Spirito

**Affiliations:** ^1^Department of Neurosciences, Reproductive Sciences and Odontostomatology, University of Naples Federico II, Naples, Italy; ^2^Department of Clinical Medicine and Surgery, University of Naples Federico II, Naples, Italy

**Keywords:** varicocele-surgery, robotic surgery, male fertility, varicocele, new technology

## Abstract

Varicocele is a pathologic dilation of the testicular veins within the spermatic cord. Varicocele is considered the most common problem in reproductive medicine practice. It is identified in 15% of healthy men and up to 35% of men with primary infertility. The exact pathophysiology of varicoceles is not very well understood, and several theories have been proposed to explain it. Varicocele can impair sperm quality and fertility *via* various mechanisms: reflux of adrenal metabolites, increased testicular hypoxia, oxidative stress, and increased testicular temperature have been proposed. Several studies have reported the significant benefits on semen parameters with the surgical treatment of varicocele: reducing oxidatively induced sperm DNA damage and potentially improving fertility. Varicocele repair should be offered as a part of treatment option for male partners of infertile couples presenting with palpable varicoceles. Nowadays, there are several surgical approaches available for the treatment of varicocele, such as the retroperitoneal approach, inguinal approach, and the subinguinal approach. The subinguinal microscopic approach offers the best outcomes, such as shorter hospital stays, preservation of the testicular arteries and lymphatics, least number of postoperative complications, recurrence, and a higher number of pregnancies. Currently robotic-assisted laparoscopic surgery is widely adopted in urology and surgeons began to explore the potential applications of the robotic platform to male infertility microsurgical operations. Robotic approach offers many advantages: elimination of tremor, retraction with third arm, high quality, 3-dimensional visualization and surgeon ergonomics, all contributing to the precision of surgery.

## Introduction

Male infertility is defined as the inability of a couple to conceive after 1 year of unprotected and frequent sexual intercourse (1). Infertility affects both men and women, with about 15% of couples that are unable to achieve pregnancy within 1 year (2). In 50% of the cases of infertility is related to male factor, while in 30% of cases, no obvious cause of abnormal sperm parameters can be found (2). Varicocele is defined as an enlargement of the pampiniform venous plexus in the scrotum and occurs in 15% of healthy men, in almost 35% of men with primary infertility, and in up to 80% of men with secondary infertility (3, 4) with a high impairment of semen parameters. Surgery and microsurgery are the most used form of treatment (5). Varicocelectomy is indicated in male infertility (impaired semen parameters or sperm quality) hypogonadism, scrotal pain, and testicular hypotrophy (5). The European Association of Urology (EAU) guidelines recommend to treat infertile men with a clinical varicocele, abnormal semen parameters, and otherwise unexplained infertility in a couple where the female partner has good ovarian reserve to improve fertility rates. A meta-analysis showed that the surgical treatment of varicocele improves semen parameters in men with abnormal semen parameters, including men with non-obstructive azoospermia, allowing a resolution of pain after surgery in 48–90% of patients (6).

Nowadays several surgical approach could be used in varicocele management and robotic approach is considered as an alternative to open or laparoscopic approach (5). Many surgeons began to use the robotic approach in microsurgical operations for male infertility due to the high quality of imagines, 3-dimensional visualization, improved surgeon ergonomics, reduction of tremor, and improved precision (7). In this review, we mainly focused on robotic role management in varicocele treatment, highlight the most relevant strength and limitation.

## Methods

We performed a narrative review on the robotic approach in varicocele treatment. Three databases (PubMed, Scopus, and ISI Web of Knowledge) were searched for articles published in English up to November September 2021 to identify studies that include robotic varicocelectomy. The following keywords were used to retrieve relevant articles: “robotic infertility” AND “” AND “robotic varicocelectomy.” Abstracts (with no subsequent full-text publications), articles that were not journal articles (letter, book, and conference proceedings), or were not peer-reviewed were excluded. Reference lists were screened for additional studies. Two authors (AA and GS) reviewed the records separately to select relevant publications, with any discrepancies resolved by a third author (BB).

## Epidemiolgy and Patophysiology

Varicocele is commonly found in male population, and it may be related to abnormal semen analysis parameters, pain and discomfort (8–10). Varicocele is found most on the left side, although there is wide variation among the reported prevalence of bilateral varicoceles, which range from 30 to 80% (8). According to recent studies, the prevalence of varicoceles in adult men is age-linked (11). Akbay et al. (12) evaluated the prevalence of varicoceles in 4,052 boys aged 2–19. They reported that the prevalence of varicoceles was <1% in boys aged 2–10, 7.8% in boys aged 11–14 years and 14.1% in boys aged 15–19 years. Oster observed that no varicoceles were detected in 188 boys 6–9 years of age but were detected with increasing frequency in boys 10–14 years of age (13). Those observations suggest that varicoceles develop at puberty and that venous incompetence, characteristic of varicocele, mainly occurs during the testicular development (14). Varicocele develops when blood flows backward into the spermatic vein causing venous dilation in the pampiniform plexus. Chehval et al. (15) studied the incidence of involvement of the external spermatic vein and discovered that 49.5% of the varicoceles had combined internal/external spermatic vein incompetence.

There are several proposed causes of blood reflux: increased hydrostatic pressure and turbulent flow caused by the perpendicular drainage of the left internal spermatic vein into the left renal vein as opposed to a more oblique inlet on the right. The course of the left internal spermatic vein results in a length of approximately 8–10 cm longer than its right-sided counterpart. This added length, coupled with upright posture, results in increased hydrostatic pressure, which can overcome valvular mechanisms in certain men and lead to the dilatation and tortuosity of spermatic veins (4). The basis for increased hydrostatic pressure and varicocele formation is best explained by Shafik and Bedeir studies. They demonstrated that patients with left varicoceles have a venous tension that is significantly higher both during rest and during Valsalva maneuver compared with that in control subjects, with average increases of 19.7 and 22 mm Hg, respectively (16). Other suggested causes include the compression exerted by the superior mesenteric artery when it crosses the left renal vein (known as the “nutcracker phenomenon”), congenital insufficient or absent venous valve. This clinical condition may affect semen quality, sperm function, testicular tissue, and reproductive hormones (16–18). Pathophysiology of varicocele-induced infertility has been widely studied and many theories have been proposed, but none has been confirmed having a leading role in testicular damage determination (17–20).

Proposed mechanisms of damage include the increase of scrotal temperature caused by varicocele may result in the deterioration of Leydig and Sertoli cell's function; an alteration of the microenvironment of the testis with an excess of renal and adrenal metabolites (21); resulting sex hormone changes; genetical predisposition; hypoxia; an increased concentration of free radicals causing oxidative stress (22). It has been observed that spermatogenesis is temperature-based and the best testicular temperature for optimal process occurs at 35–36°C. Varicocele related reversal venous blood flow may increase scrotal temperature by approximately 2.5°C (23). Chronic testicular exposure at higher temperature causes heat stress with impaired testis function, as it has been demonstrated with artificially induced cryptorchidism (24). Several hypotheses related to the pathophysiology of varicocele are derived from studies carried out through the use of animal models. Sofikitis and Migawa highlighted the harmful effects of varicocele on the testicles of rabbits and how the seminal parameters improved after surgery. These results are also associated with observations in the rat model there is a cause-effect relationship between a persistent primary varicocele and a right secondary varicocele, since no operated rabbit developed a secondary right varicocele (25, 26).

Moreover, it has been shown that the rat varicocele model induces reactive oxygen and apoptosis species in the testicles, through an increased expression of the Bax protein, which is a pro-apoptotic protein (27).

The role of oxidative stress has been widely studied since it is normal for sperm cells to produce a certain amount of reactive oxygen species (ROS). ROS have a physiological role in permitting functions, such as fertilization or capacitation. Due to the abundance of polyunsaturated fatty acids in the spermatozoa membrane, an excess of seminal ROS levels may lead to lipid peroxidation thus compromising the sperm cell motility. The fertilizing level of sperm cells may be also harmed by DNA damage caused by the high levels of seminal ROS (28). However, although seminal markers of oxidative stress are increased in fertile men with varicoceles, this does not result in a deterioration of reproductive capacity. It has been hypothesized that variations in genetic transcriptional response to oxidative stress could confer sperm protection against damage and thus explain why some men with varicocele retain their reproductive potential. Another hypothesis holds that an increase in the extracellular testicular fluid (testicular extracellular edema) determine the testicular consequences of left varicocele and therefore patients with an adequate testicular lymphatic drainage system have an unaltered testicular function (29, 30).

Moreover, although there is evidence that support a beneficial effect of varicocele treatment on oxidative-stress-associated infertility, is unclear why fertility does not improve in all patients undergoing varicocelectomy (31).

Regarding the role of genetic factors involved in the development of varicocele, many hypotheses have been made. However, the routine uses of specific gene sequences, mutation analyses, and studying the sperm epigenome are nowadays not recommended, due to the multifactorial nature of varicocele, the cost, the availability of equipment, and the low clinical relevance (22).

## Surgical Technique

Several surgical techniques are available in varicocele repair, such as inguinal and subinguinal open ligation, laparoscopic retroperitoneal ligation, and transvenous occlusion. The best solution should be characterized by the ligation of the internal spermatic venous drainage of the testicle while preserving arteries and lymphatics (32).

## Subinguinal Varicocelectomy

Subinguinal varicocelectomy consists of a 3-cm transverse skin incision over the external inguinal ring until it reaches the Scarpa fascia that is dissected using the index finger. The cord structures are grasped with a Babcock clamp and then secured by an army-navy retractor or a Penrose drain. With the aid of a surgical microscope, the external spermatic fascia is incised and all veins within the spermatic cord are ligated with 4-0 ties. Compared with this technique, the inguinal approach is associated with increased postoperative pain because of the opening of the external oblique fascia (33). In this case, the incision is made over the lower inguinal canal, starting two finger breadths medial and caudal to the anterior iliac spine. After incision, the aponeurosis of the external oblique fascia, the spermatic cord is isolated and divided, taking care not to damage the ilioinguinal nerve and testicular arteries (34). The veins are then ligated (35).

## Laparoscopic Varicocelectomy

Laparoscopic varicocelectomy is performed with the use of three transperitoneal ports. The two most common techniques are the non-artery-sparing laparoscopic varicocelectomy and the artery-sparing laparoscopic varicocelectomy. In the first, the testicular artery and spermatic veins are ligated with the higher rates of varicocele recurrence, whereas in the second only, the veins are ligated with a high risk of hydrocele (36, 37). In both approaches, the peritoneum is incised approximately 3 cm superior to the internal inguinal ring to gain access to the gonadal vessels.

## Robotic-Assisted Microscopic Varicocelectomy

This procedure starts with an incision of 3 cm from the base of the penis and 3 cm lateral to the midline. The spermatic cord is isolated through the incision using 3.5 × loupe magnification. A Penrose drain could be used to suspend the spermatic cord extracorporeally. In the case of bilateral varicocele, both cords are isolated before the robot docking. The robot is docked at the left side of the patient. The first arm is aligned with the right anterior superior iliac spine toward the incision; the second arm is 90 degrees from the first arm between arms one and three; the third arm is aligned with the ipsilateral knee to the incision. Two black diamond forceps are generally used and loaded on arms one and three. The second arm is loaded with monopolar scissors. A zero degree lens camera is used. The first step is opening the cremasteric fascia exposing the vas deferens that is isolated with a vessel loop. The testicular artery is also isolated with a second vessel loop. Sometimes papaverine could be used to enhance pulsations to clearly identify the arteries. Then, veins are dissected, ligated with titanium clips, and separated. At the end, the cremasteric fascia is usually reconstructed with 8-0 nylon suture.

## Robotic-Assisted Laparoscopic Varicocelectomy

Robotic-assisted laparoscopic varicocelectomy (RALV) requires the establishment of a pneumoperitoneum (12 mm Hg). Three trocars are used. The first one is a 10 mm Trocar, placed as the camera port. Other two 5 mm trocars are placed in lower quadrants approximately 8 cm away from the camera port. A zero degree lens camera is used. Different instruments could be loaded on the arms depending on surgeon preferences. The first step is the identification of the spermatic cord. Then, the overlying retroperitoneum is incised proximally to the internal inguinal ring. Spermatic vessels are accurately dissected. Arteries and veins are isolated, indocyanine injection could be used for the identification. All the veins are ligated, sparing arteries and lymphatics. Testicular artery sparing is one of the advantages of RALV. Preserving TA during varicocelectomy or not, remains controversial. Theoretically, the preservation of TA guarantees optimal blood supply to the testis.

## Discussion

Since the introduction of the first operative microscope in male infertility procedure in 1970, several developments have occurred. The da Vinci surgical system is the only commercially available robotic platform, widely used for several types of surgical procedures (38). Recently some studies showed robotic use in microsurgical procedures for male infertility included varicocele treatment (39, 40). Subinguinal microscopic varicocelectomy showed superior outcomes compared with other techniques (41). Al-Kandari et al. (42) in a randomized clinical trial comparing the functional outcome of three different surgical techniques of varicocelectomy (open inguinal approach, laparoscopic approach, and subinguinal microscopic varicocelectomy) showed better outcomes with subinguinal microscopic varicocelectomy in the terms of recurrence and adverse effect, while the pregnancy rate and improvement in sperm parameters were comparable (65, 67, and 76% of the open, laparoscopic, and microscopic groups, respectively). In addition, the subinguinal approach allows for testicular delivery, minimizing the risk of recurrence thanks to the identification and ligation of collateral veins, such as the external pudendal, external spermatic (cremasteric), and gubernacular veins (43).

As gubernacular veins, found in 71–79% of cases, are held responsible for postoperative relapses, some authors suggest that microsurgical varicocelectomy should include delivery of the testis to ligate the gubernacular veins. However, according to Ramasamy and Schlegel, there are no significant differences in terms of recurrence after the ligation or non-ligation of gubernacular veins by comparing varicocelectomy with and without the delivery of testis (44, 45).

In another study, Cayan et al. (46) showed that among different surgical approaches, the sub-inguinal microsurgical varicocelectomy has higher spontaneous pregnancy rates (41.97 vs. 30.07% in the laparoscopic, 33.2% radiologic embolization, and 36% macroscopic inguinal, *p* = 0.001), lower postoperative recurrence (2.63 vs. 4.3% in the laparoscopic, 12.7% radiologic embolization = 0.001), and lower complication rates when compared with other techniques.

The laparoscopic approach shows the advantage of isolating internal spermatic veins near the left renal vein so the recurrence rate is very low, and the testicular artery can be preserved (3.5–20%). The most important complications in laparoscopic varicocelectomy are air embolism, inadvertent arterial division, genitofemoral nerve injury, hydrocele, intestinal injury, and peritonitis occurring in 8–12% of cases. The limitations of this technique are: high cost, need of general anesthesia, and days of hospitalization (5). Robotic surgery showed several advantages concerning other techniques: elimination of tremor, retraction with the third arm, high quality, 3-dimensional visualization, and surgeon ergonomics, all contributing to the precision of surgery (47). Shu et al. (48) described one of the earliest robotic-assisted microscopic varicocelectomy (RAMV) in comparison with traditional microscopic varicocelectomy (TMV). RAMV was more safe and effective than microscopic surgery. The operating times were similar (73.9 ± 12.2 vs. 71.1 ± 21.1), while there was a benefit in eliminating tremors, decreased intraoperative and postoperative complications. Additionally, McCullough et al. (49) reported that RAMV was a safe and effective alternative for varicocele repair than a microsurgical approach. Data showed increased sperm concentration of 37.3% (*p* < 0.03), testicular left and right volume increased to 22.3% (*p* < 0.0001) and 12.6% (*p* < 0.0006), respectively (49).

Parekattil et al. (41) reported in their case series that 77% of patients treated with RAMV showed an improvement on sperm count (18% were azoospermic and become oligospermic) or motility and a reduction of testicular pain/orchialgia (85% of patients). Table 1 describes the advantages and disadvantages of different surgery technique.

**Table 1:** Advantages and disadvantages of different surgical approaches.

	**Open**	**Laparoscopic**	**Robotic**
Advantages	Touch enhanced Local anesthesia Lower cost Lower procedure time Lower risk of hydrocele	Shorter hospitalization Faster postoperative recovery Lower recurrence rate Lower risk of testicular atrophy Most sense for bilateral cases	Seven degrees of freedom Tremor elimination 3D visualization Better vessel identification Better cosmesis Surgeon ergonomics
Disadvantages	Tremor amplified Less sterile Longer hospitalization Pain Higher risk of recurrence	Long Learning Curve General anesthesia Increased risk of vascular or visceral injury Higher risk of hydrocele	Learning curve Touch absense Cost General anesthesia required Higher procedure time

Robotic varicocelectomy shows several limitations: first, the cost of robotic surgery is much higher than other approaches. Second, external spermatic perforators/gubernacular veins could not be handled under a robotic laparoscopic approach (39, 50).

Second, microsurgeons were generally skeptical regarding the delicate tissue handling capabilities of the robotic approach (50) and case series were limited and no randomized controlled trials were available. Additionally, although early studies suggest relatively quick learning with the robotic approach, surgeon experience and comfort with the traditional microscope limit the wide adaptability of the robot (40). Another problem is related to the costs of the robotic platform and the annual maintenance and disposable costs procedure, and this is the cause of substantial drawback of robotic surgery. Robotic surgery involves indirect costs, as mentioned previously, that seem to be the main obstacle in economic terms and direct costs (room and board, anesthesia, operative room expenses, etc.) (51).

Instead, in the case of the acquisition of the operating microscope, the cost is reduced, as are virtually non-existent costs related to its use.

According to Parekattil et al. (41) the only solution to reduce the costs of the robotic approach would be to drastically increase the number of procedures.

Although when reproductive urologists operate within a healthcare system already in the possession of a surgical robot, the added use costs could be substantially less.

In conclusion, robotic assistance for microsurgical procedures in male infertility is safe and feasible. The advantages include an elimination of tremor, multi-view magnification, additional instrument arms, a short learning curve with small skin incisions (41). This paper gives an overview of robotic varicocele approach based on the relevant article published in recent years, but has several limitation, first of all is a narrative review and we did not evaluated the quality of the studies included and the case series is very small.

However, prospective, randomized, and comparative studies with many patients are needed to compare the efficacy of robotic varicocelectomy with other treatment modalities in infertile men. The reduction of costs and the availability of robotic platforms will certainly lead to an increase in the robotics approach in male infertility.

## Author Contributions

LS: conceptualization. LN, RL, SP, AA, and GS: methodology. LN, LS, GM, BB, and CR: formal analysis. GC, RM, LC, LN, and LS: resources. LN, SP, AA, GS, LS, and LC: writing-review and editing and writing-original draft. LN, LS, RL, and CR: supervision. All authors contributed to the article and approved the submitted version.

## Conflict of Interest

The authors declare that the research was conducted in the absence of any commercial or financial relationships that could be construed as a potential conflict of interest.

## Publisher's Note

All claims expressed in this article are solely those of the authors and do not necessarily represent those of their affiliated organizations, or those of the publisher, the editors and the reviewers. Any product that may be evaluated in this article, or claim that may be made by its manufacturer, is not guaranteed or endorsed by the publisher.
